# Evaluation of the Cyclic Fatigue of Two Single Files at Body and Room Temperature with Different Radii of Curvature

**DOI:** 10.3390/ma14092256

**Published:** 2021-04-27

**Authors:** Giusy Rita Maria La Rosa, Valeria Shumakova, Gaetano Isola, Francesco Indelicato, Calogero Bugea, Eugenio Pedullà

**Affiliations:** 1 Department of General Surgery and Medical-Surgical Specialties, University of Catania, Plebiscito 628, 95124 Catania, Italy; valve6@yandex.ru (V.S.); gaetanoisola@gmail.com (G.I.); indelicato@policlinico.unict.it (F.I.); eugeniopedulla@gmail.com (E.P.); 2 Private Practice, Vespucci 10, 73044 Lecce, Italy; calogerobugea@yahoo.it

**Keywords:** body temperature, cyclic fatigue resistance, F6 SkyTaper, One Curve, radius of curvature

## Abstract

**Background:** To compare the influence of different temperatures and curvature radii on the cyclic fatigue resistance of F6 SkyTaper (F6ST) and One Curve (OC) single file nickel-titanium rotary instruments. **Methods:** A total of 120 instruments of F6ST and OC #25.06 were evaluated in 5 mm and 3 mm curvature radii at two temperatures (20 °C ± 1 °C and 37 °C ± 1 °C) in 16 mm stainless steel artificial canals associated with a curvature of 60°. The cyclic fatigue of tested files was assessed by employing a customized testing apparatus and expressed as times to fracture (TtF). A statistical analysis was performed with the significance level set at 95%. **Results:** All instruments decreased their TtF at 37 °C except for OC in the 3 mm radius, in which no significant difference was detected between 20 °C and 37 °C. A 3 mm curvature radius negatively affected TtF of all tested instruments, except for F6ST at 20 °C. F6ST had higher TtF than OC in the 3 mm radius at 20 °C, with no significant difference between them in the other tested conditions. **Conclusions:** Under the limits of the present in vitro study, body temperature impaired cyclic fatigue resistance of all files, except for OC in the 3 mm curvature radius. All instruments exhibited lower times to fracture in the 3 mm radius, excluding F6ST at 20 °C.

## 1. Introduction

Nickel-titanium (NiTi) instruments possess advantageous properties, such as extreme flexibility and the elasticity necessary for safe root canal preparation, especially in the presence of accentuated curvatures [1,2]. Nevertheless, an important limitation of NiTi files is the possibility of unexpected breakage during use in root canals due to cyclic fatigue or torsional fatigue [3,4]. In cyclic fatigue, a specific area of the NiTi instrument is repetitively exposed to cycles of compression and tensile forces which could lead to a fracture [5]. Numerous elements affect the flexibility of NiTi instruments, such as anatomical configurations, file design including the cross-sectional area, surface, and metallurgical treatments [6]. Among the anatomical variables implicated in instrument separation, the radius of curvature assumes a pivotal role [7,8]. In particular, a small radius is associated with higher flexural stresses [7,9]. In order to enhance the cyclic fatigue of instruments, thermomechanical treatments have been proposed for NiTi alloys. Thermomechanical manufacturing refines the structure and transformation performance of the file, influencing the physical behavior of the instrument [10,11]. In addition, root canal preparation techniques have evolved during the last few years in order to simplify the procedure and reduce the number of files used. In this context, single file systems have been developed [12]. F6 SkyTaper (Komet, Brasseler GmbH & Co., Lemgo, Germany) is a single-file system constituted by the traditional austenite 55-NiTi alloy. All files exhibit a continuous taper of 0.06 for the whole working part with a modified S-shaped cross-section [13,14]. One Curve (Micro-Mega, Besançon, France) is also a single file rotary made of a C-Wire obtained through an innovative heat treatment process which should improve the file flexibility. The One Curve presents only one file (25.06) possessing a changeable cross-sectional design [15,16]. Although many previous investigations on cyclic fatigue have been performed at room temperature [11,17], body temperature (around 37 °C) may significantly affect the transformation temperatures of NiTi crystalline phase and thus the cyclic fatigue behavior of NiTi files [1,18,19].

Only one previous study evaluated the effects of body and room temperatures associated with different angles of file inclination on the cyclic fatigue resistance of OC and F6ST [20]. Nevertheless, in that study, the influence of an external factor (i.e., file inclination), potentially modifiable, was evaluated. Currently, no studies have assessed the simultaneous effect of temperature and radius of curvature on cyclic fatigue resistance of F6 SkyTaper and One Curve single files. Therefore, the purpose of the present study was to investigate the cyclic fatigue resistance of two single file nickel titanium rotary instruments (F6ST and OC) at different temperatures and curvature radii using a customized testing apparatus which ensured standardized methodological conditions. The null hypothesis was there would be no difference between the tested files with different radii of curvature at room (20 °C ± 1 °C) and body (37 °C ± 1 °C) temperatures.

## 2. Materials and Methods

**Figure 1:**
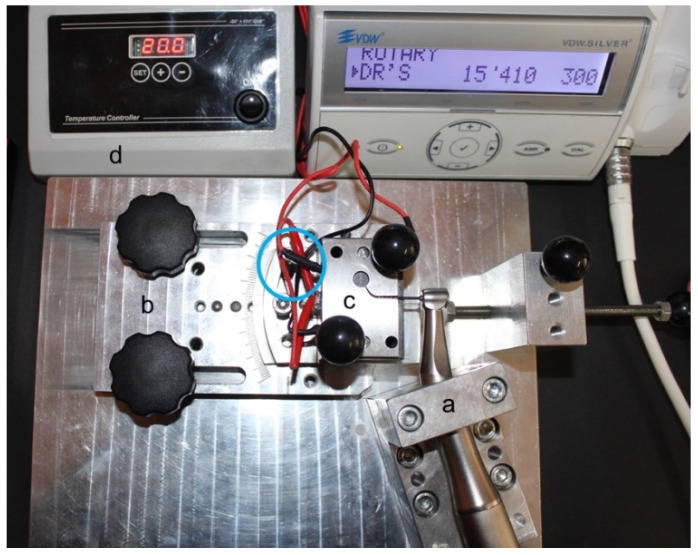
The customized cyclic fatigue apparatus with an OC #25.06 file at 20 °C in the 5 mm curvature radius. (**a**) The block system maintained the electric handpiece in a fixed position; (**b**) the movable support on rails guaranteed to insert/remove the instrument always in the same manner; (**c**) the interchangeable plate contained the artificial canal with modifiable radii and angles of curvature; (**d**) the thermostat employed to check the temperature variation in the testing canal. The blue circle indicates the thermocouple used in the artificial canal.

Sample size determination was obtained through a pilot study in which a test power of 0.80 was established (G*Power 3.1.9.2 software, Heinrich-Heine-Universität Düsseldorf, Düsseldorf, Germany) with α = 0.05. Under these conditions, at least 15 files for each group (*n* = 15) were indicated as necessary to detect significant differences. Consequently, 120 new files (#25. 06 F6ST and OC, 25 mm long) were selected. Sixty files of each brand (F6ST and OC) were tested in two different artificial stainless steel canals presenting the same curvature angle (60°), but two different radii curvatures of 5 and 3 mm, respectively. Instruments (*n* = 15) were tested in each canal at 2 different temperatures (20 °C ± 1 °C and 37 °C ± 1 °C) (Figure 1).

Instruments with hypothetical visible deformations were identified by a preliminary assessment of their surface through an optical stereomicroscope with 20× magnification (SZR-10; Optika, Ponteranica, Bergamo, Italy). No instrument was excluded.

### 2.1. Cyclic Fatigue Testing Device

**Figure 2:**
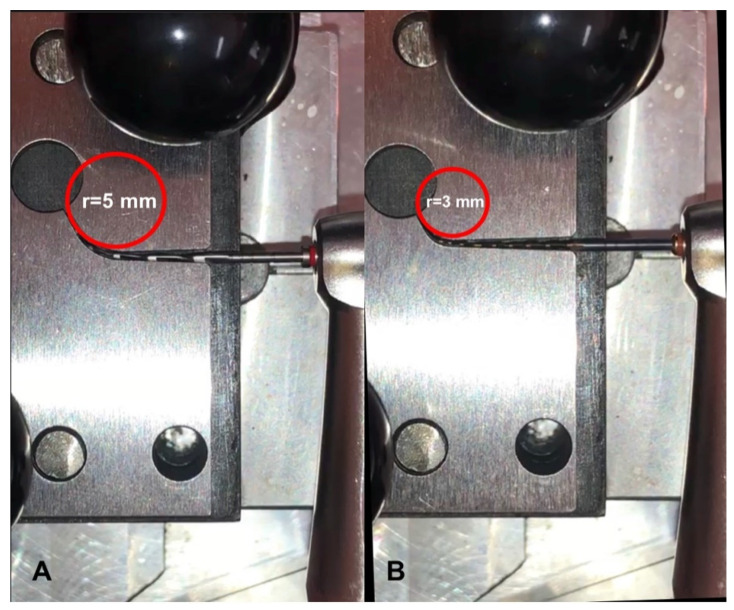
The artificial canals employed in the current study. F6 SkyTaper (**A**) and One Curve (**B**) were located in the testing canal in (**A**) 5 mm radius or (**B**) 3 mm radius canals.

A customized testing apparatus, previously described [8,20,21,22], was employed to perform cyclic fatigue resistance tests. Briefly, it ensured the 6:1 reduction electric handpiece (Sirona Dental Systems GmbH, Bensheim, Germany) was maintained at a constant three-dimensional location through a fixed block. Furthermore, a movable support on rails allowed us to insert/remove the instrument always in the same standardized manner. A blockage system maintained the movable support at the same location. The electric handpiece allowed us to insert each file into the artificial canal in an exact and replicable manner (Figure 2).

The artificial canal was explicitly planned for the corresponding file referring to the size and taper, providing an adequate lumen with suitable trajectory [23]. Moreover, it was possible to test instruments with different inclinations to mimic clinical situations, keeping the file insertion always perpendicular to the canal. In the present study, all files were assessed at the standard position of 0°, corresponding to an ideal straight access of file into the canal [8,20,21,22]. To check the temperature variation in the artificial canal, a thermostat was linked with the customized device. The temperature was maintained at a constant value during the test by the means of a thermocouple located in the artificial canal which turned on or off the thermostatic resistance when the temperature reached less or higher values than the predetermined one, respectively [20,22]. All tested files were switched on at the predefined temperature by means of the handpiece driven by a torque-controlled motor (Silver Reciproc, VDW, Munich, Germany) in continuous rotation at a constant speed of 300 rpm (revolutions per minute) and the maximum torque (4.1 Ncm) as suggested by the manufacturer. Each file was inserted in the contra-angle handpiece and then positioned into the testing canal always in the same way. A specific high-flow synthetic oil (Super Oil; Singer Co Ltd., Elizabeth, NJ, USA) was applied to decrease the file friction on the artificial canal walls. The rotation of each file remained active up to the breakage. In addition, a digital caliper (Digimatic; Mitutoyo Co, Kawasaki, Japan) was used to determine the length of the fractured tip. Cyclic fatigue resistance was expressed as time to fracture in seconds (TtF) from the beginning of the test until the moment of fracture. TtF was monitored visually and/or audibly using a chronometer with an accuracy of 0.1 s. Moreover, video recording was concurrently executed and checked to confirm the time previously recorded and to avoid human errors.

### 2.2. Statistical Analysis

Normality of data was evaluated by the Shapiro-Wilk test. Once their normality was confirmed, the data were analyzed by using 2-way ANOVA and the Bonferroni multiple comparison post hoc test (Prism 8.0; GraphPad Software, Inc, La Jolla, CA, USA). Statistical significance was set at 95% (*p* < 0.05).

## 3. Results

The mean ± standard deviation values referred to the cyclic fatigue resistance in TtF (sec) for each environmental condition are reported in Table 1.

Regarding temperature, all tested files decreased their TtF at 37 °C (*p* < 0.05) in both radii of curvature, except for OC in the 3 mm radius, in which no significant difference emerged between 20 °C and 37 °C (*p* > 0.05). As for radius of curvature, all instruments decreased their cyclic fatigue resistance in the 3 mm radius in comparison with the 5 mm radius (*p* < 0.05) at both temperatures, except for F6ST at 20 °C in which there was no significant difference between the two radii of curvature (*p* > 0.05). Comparing the two instruments, F6ST showed higher TtF than OC at 20 °C in the 3 mm radius (*p* < 0.05), while no significant difference resulted between them in all other tested conditions (*p* > 0.05).

In addition, no significant difference was reported as a concern for the mean length of the fractured fragments for any of the instruments tested (*p* > 0.05). More specifically, a length of 5 ± 0.1 mm was recorded for instruments activated in the 5 mm radius while 3 ± 0.2 mm was recorded for files rotated in the 3 mm radius.

## 4. Discussion

This study aimed to assess the fatigue life of two single file systems tested at 3 and 5 mm radii of curvature at room and body temperatures. An in vitro simulation offers a simplified reproduction of the clinical practice regarding anatomical conditions of canal and temperature at which tests are performed [19]. The current customized device ensured the assessment of cyclic fatigue behavior of NiTi endodontic files under different radii of curvature and temperature conditions, obtaining reproducible tests not operator-dependent [8,20,21,22]. Indeed, the same and reproducible file insertion into the artificial canal was confirmed by the point of the fracture of broken segments always reported at the center of curvature tested [24,25]. Radii of curvature of 3 mm and 5 mm were selected because they represent the most frequent values reported for apical curvature in some previous studies [7,8,26,27]. Temperature was set at 20 °C ± 1 °C and 37 °C ± 1 °C to reach a valid simulation of room and body temperature conditions [16]. In the current study, F6ST and OC were selected because they are two single-file NiTi instruments that are both available in size 25.06 and very common among clinicians that differ in cross-sectional design and heat treatment. Indeed, F6ST instruments are made with a traditional austenite 55-NiTi alloy with a modified S-shaped cross section along their entire working part [13,14,28]. OC files are produced with a novel heat treatment and possess, according to the manufacturer, a variable design which progressively changes from S-shaped design close to the shaft to a triangular cross section in the apical middle part, including at 5 mm and 3 mm as previously reported [11,15,16]. Although a dynamic model seems to mimic clinics better compared to the static one, it is challenging to guarantee repeatable and standardized laboratory conditions for each instrument. Therefore, in the current study, a static model was used to exclude any other confounding factor apart from cyclic fatigue [9,29]. As previously reported, the fatigue life was expressed in time and not in the number of cycles to failure because time is much more straightforward and reliable for the clinicians [21,30].

According to our results, body temperature impaired fatigue life of all tested files except for the OC in 3 mm radius of curvature in which no significant difference emerged between the tested temperatures. Therefore, the null hypothesis was partially rejected. To clarify the significant decrease in cyclic fatigue resistance at body temperature, one may consider how surrounding temperature affects NiTi crystalline phase transformation, also as concerns for the previous heat treatment to which NiTi alloy has been subjected [16,17,19]. NiTi alloy is characterized by two heat-dependent crystal states (martensite and austenite), and the metal properties vary in each of these phases. It is more rigid in austenitic phase at temperatures higher than the transformation level and, successively to thermic or mechanical inputs, can modify into a martensite phase, which makes it more flexible [11,24,31]. Generally, instruments made by traditional NiTi alloys (such as F6ST) [13,28] possess an austenite finish temperature (A_f_) under the body temperature [31] while heat treated instruments such as OC exhibit A_f_ temperature higher than the body temperature (for OC 40–50 °C) [24]. For both instruments, the passage from 20 °C to 37 °C induces the progressive shifting to an austenite phase causing a progressive reduction in the lifespan of the NiTi files. On the basis of these results, it is reasonable to think that this difference was not significant for OC in the 3 mm curvature radius because the abrupt apical curvature had a major impact on the flexural stress of instruments compared with temperature, reducing the times to fracture both at 20 °C and 37 °C. Consequently, even if body temperature reduced the TtF of OC files, the difference between the two temperatures was not sufficient to reach the significance level.

In agreement with the previous studies [7,8,9,32], the smaller the curvature radius is, the greater the flexural stress of the NiTi file. Only for F6ST at 20 °C there was no significant difference between 3 mm and 5 mm radii of curvature. These results could be attributed to the reduced core area of the S-shaped cross-sectional design [14]. The benefits provided by this cross-sectional design on the cyclic fatigue resistance is also demonstrated by some previous authors [7,33]. This advantage was not significant at 37 °C, probably due to the fact it was compensated by the significant negative impact of increase of temperature. Therefore, on the basis of these results, it could be speculated that the cyclic fatigue of OC files, heat treated and with a triangular cross section at the apical level [15,16], is more affected by the small radius of curvature, while F6ST instruments made by traditional NiTi alloy and with an S-shaped cross section [13,14] are more influenced by temperature.

Comparing the two instruments, F6ST exhibited significantly higher TtF than OC at 20 °C in the 3 mm radius of curvature, with no difference between them in the other experimental setting. This result is presumably due to the benefit provided by the S-shaped cross section design of F6ST in the most abrupt curvature of 3 mm radius at room temperature.

To the best of our knowledge, this is the first study comparing the effects of different radii curvature at body and room temperatures on OC and F6ST cyclic fatigue using a customized apparatus to ensure standardized laboratory procedures. Indeed, as previously mentioned, only one study assessed the influence of an inclined file access at different temperatures on cyclic fatigue resistance of F6ST and OC files [20]. Nevertheless, the file inclination (similar to a further external curvature) is a condition different from the apical curvature of root canal. Therefore, a direct comparison with those results is not possible. However, both studies showed different environmental conditions could affect the lifespan of NiTi instruments in a different manner.

Thus, it is important for clinicians to select the appropriate NiTi rotary instrument according to anatomical variables involved as complex curvatures [26,34]. Within the limitations of an in vitro study and instruments included, this investigation offers to the clinicians an indication on how cross sectional design and metallurgical features act simultaneously in affecting the behavior of files into the root canal. Caution is necessary to apply the current results in the clinical environment. Indeed, intracanal instrumentation is affected by numerous elements such as torsional stress [21,23], anatomical complexities, the type of instrument (i.e., geometrical and metallurgical features), and an inclined file insertion into the canal [20,21,35]. All these factors perform simultaneously and can affect flexural behavior of NiTi files. Future clinical laboratory evaluation on the crystalline phase of different NiTi alloys at different environmental conditions could be of interest to determine the extent to which NiTi files might be expected to be more flexible, and to help select the best instrument according to the clinical conditions. Future in vivo research will have to confirm the present findings.

## 5. Conclusions

Under the limits of this laboratory study, it can be concluded that body temperature impaired cyclic fatigue resistance of all files, except for OC in the 3 mm curvature radius. All tested files exhibited lower times to fracture in the 3 mm radius apart of F6ST at 20 °C. No significant difference was reported between the two instruments tested except at 20 °C in the 3 mm radius in which F6ST performed significantly better than OC.

## Figures and Tables

**Table 1:** The time to fracture (Mean ± Standard Deviation) in seconds of F6 SkyTaper and One Curve in 5 mm and 3 mm curvature radii tested at room (20 °C ± 1 °C) and body (37 °C ± 1 °C) temperatures.

	**Temperature**			
	**20 °C**		**37 °C**	
	**Radius of Curvature**			
	**5 mm**	**3 mm**	**5 mm**	**3 mm**
**Instrument**	**Time to Fracture (sec)**			
**F6 SkyTaper**	195 ^a1^ ± 30	171 ^a1^ ± 27	149 ^b1^ ± 27	90 ^c1^ ± 31
**One Curve**	175 ^a1^ ± 28	115 ^b2^ ± 26	128 ^c1^ ± 26	105 ^b1^ ± 28

Different superscripts indicate a significant difference between instruments in the same row (*p* < 0.05). Different superscript numbers indicate a significant difference between instruments in the same column (*p* < 0.05).

## Data Availability

The data presented in this study are available on request from the corresponding author.
